# Efficacy and safety of *Ojeok-san* in Korean female patients with cold hypersensitivity in the hands and feet: study protocol for a randomized, double-blinded, placebo-controlled, multicenter pilot study

**DOI:** 10.1186/s13063-018-3013-9

**Published:** 2018-11-29

**Authors:** Youme Ko, Ho-Yeon Go, In-Sik Han, Kyou-Young Lee, Tae-Hoon Kim, Jin-Moo Lee, Jun-Bok Jang, Yun-Kyung Song, Seung-Ho Sun, Chan-Yong Jeon, Seong-Gyu Ko

**Affiliations:** 1 Department of Korean Preventive Medicine, Graduate School, Kyung Hee University, 26 Kyungheedae-ro, Dongdaemun-gu, Seoul, Republic of Korea; 2 Department of Korean Internal Medicine, College of Korean Medicine, Semyung University, 65 Semyeong-ro, Jecheon-si, Chungcheongbuk-do Republic of Korea; 3 Department of Korean Internal Medicine, College of Korean Medicine, Sangji University, 80 Sangjidae-gil, Wonju-si, Gangwon-do 26339 Republic of Korea; 4 Department of clinical trial center, College of Korean Medicine, Kyung Hee University Hospital, 23 Kyungheedae-ro, Dongdaemun-gu, Seoul, Republic of Korea; 5 Department of Korean Gynecology, College of Korean Medicine, Kyung Hee University, 26 Kyungheedae-ro, Dongdaemun-gu, Seoul, Republic of Korea; 6 Department of Korean Rehabilitation Medicine, College of Korean Medicine, Gachon University, 1342 Seongnamdae-ro, Sujeong-gu, Gyeonggi-do Republic of Korea; 7 Department of Korean Internal Medicine, College of Korean Medicine, Gachon University, 1342 Seongnamdae-ro, Sujeong-gu, Gyeonggi-do Republic of Korea

**Keywords:** Herbal medicine, Cold temperature, Cold hypersensitivity, Randomized clinical trial, Korean medicine

## Abstract

**Background:**

This study aims to evaluate the safety, efficacy, and feasibility of a full randomized clinical trial of *Ojeok-san* in Korean female patients with cold hypersensitivity in the hands and feet.

**Methods:**

This study is a multicenter, double-blinded, randomized, placebo-controlled, two-arm, parallel-group pilot clinical trial. A total of 60 participants will be enrolled and randomly assigned to the *Ojeok-san* treatment group or the placebo control group, in a 1:1 ratio using an Internet-based randomization system. Each group will be administered *Ojeok-san* or placebo three times per day for 8 weeks. The primary outcome will be the mean change in the Visual Analog Scale scores of cold hypersensitivity in the hands from baseline to week 8. Secondary outcomes will include the mean changes in the skin temperature of the extremities, recovery rate of the skin temperature of hands after cold stress test, and the score of Korean version of the WHO Quality of Life Scale abbreviated version.

**Discussion:**

The findings of this study should provide meaningful information for a further large-scale, randomized controlled trial to confirm the safety and efficacy of *Ojeok-san* on cold hypersensitivity in the hands and feet in female patients.

**Trial registration:**

ClinicalTrials.gov, ID: NCT03083522. Registered on 20 March 2017.

**Electronic supplementary material:**

The online version of this article (10.1186/s13063-018-3013-9) contains supplementary material, which is available to authorized users.

## Background

Cold hypersensitivity in the hands and feet (CHHF) is a common complaint in female patients in East Asian countries. It is defined as a subjective symptom in which the individual feels strong coldness in the extremities compared with other individuals under any environmental conditions [1, 2]. According to the Comprehensive Survey of Living Conditions 2013, 32.6% of Japanese women have a complaint of CHHF compare to 15.3% of men [3]. The prevalence of CHHF in Korea has been reported as approximately 12%, with greater effect on the quality of life in the Korean female as compared to the Korean male population [4].

In Korea, the general structure of the healthcare system is pluralistic, providing conventional and traditional Korean medicine based on the patient’s choice. It has the advantage of offering a broad range of medical support to patients based on their own decision [5]. However, due to the lack of evidence on the etiology of CHHF, the standardized clinical practice guideline on CHHF is not yet fully developed, and may provide less certainty and positive expectations of improvement to patients.

Currently, the conventional medicine (CM) perspective on the mechanism of CHHF includes the reduction of peripheral blood flow in the extremities or the increase in peripheral vasoconstriction during exposure to cold temperatures such as, Raynaud’s phenomenon (RP). The common treatment plans of RP consider alleviating each accompanying symptoms itself, including vasodilators, calcium-channel blockers, and lifestyle modifications, but the therapeutic effects of such treatments are debatable [6, 7].

However, traditional Korean medicine (TKM) offers a different perspective on CHHF from ancient times. Based on the diagnostics of TKM, CHHF has been classified as a cold-related symptom as well as a cold syndrome, which requires careful attention to prevent symptom aggravation and further pattern progression into a severe condition [8]. This syndrome is generally categorized into three types by different pathogens. The first type is the invasion of external pathogen of cold through the mouth or body surface, and affects the whole-body condition. The second type is the disharmony of specific internal organs, such as spleen *yang* deficiency, kidney *yang* deficiency, blood deficiency, or *Qi* deficiency. The third type is simultaneous occurrence of cold and heat syndrome due to loss of stomach harmony and lowering adverse *Qi* [1, 9].

The traditional herbal formula, *Ojeok-san* (OJS; *Wuji-san* in Chinese, *Goshaku-san* in Japanese) has originated from the traditional Chinese medicine (TCM) classic *Prescriptions from the Great Peace Imperial Grace Pharmacy* by Chen Shiwen [10]. OJS is known as a general prescription that treats pathogen accumulation due to internal and external invasion by pathogenic factors, such as cold, *Qi*, blood, phlegm, and food. Based on the TKM literature review on OJS, the main indications of OJS are pain in various body parts, gynecological and gastrointestinal symptoms, such as whole-body pain, back pain, CHHF, vomiting, and diarrhea [11–13]. OJS has been widely used in traditional herbal prescriptions, accounting for approximately 30% of drug utilization among the list of National Health Insurance (NHI)-covered herbal preparations in 2011 in Korea [14]. The TKM expert survey on the epidemiology and treatment of CHHF also has indicated that OJS was one of the frequently prescribed medication for CHHF treatment in the clinical field [1, 2].

Despite evidence from TCM and TKM literature and clinical consensus on OJS as a CHHF treatment, a clinical trial to assess the efficacy and safety of OJS has not yet been conducted in any East Asian population. Thus, a well-designed, high-quality, multicenter, randomized, double-blinded, placebo-controlled trial is needed to provide more evidence for the treatment of patients with CHHF using herbal medicine.

## Methods

### Objective

We will evaluate the feasibility of a full randomized clinical trial (RCT) of OJS on Korean female patients with CHHF to evaluate CHHF symptom reduction after 8 weeks of OJS medication as compared with the placebo group.

### Trial design and setting

**Fig. 1:**
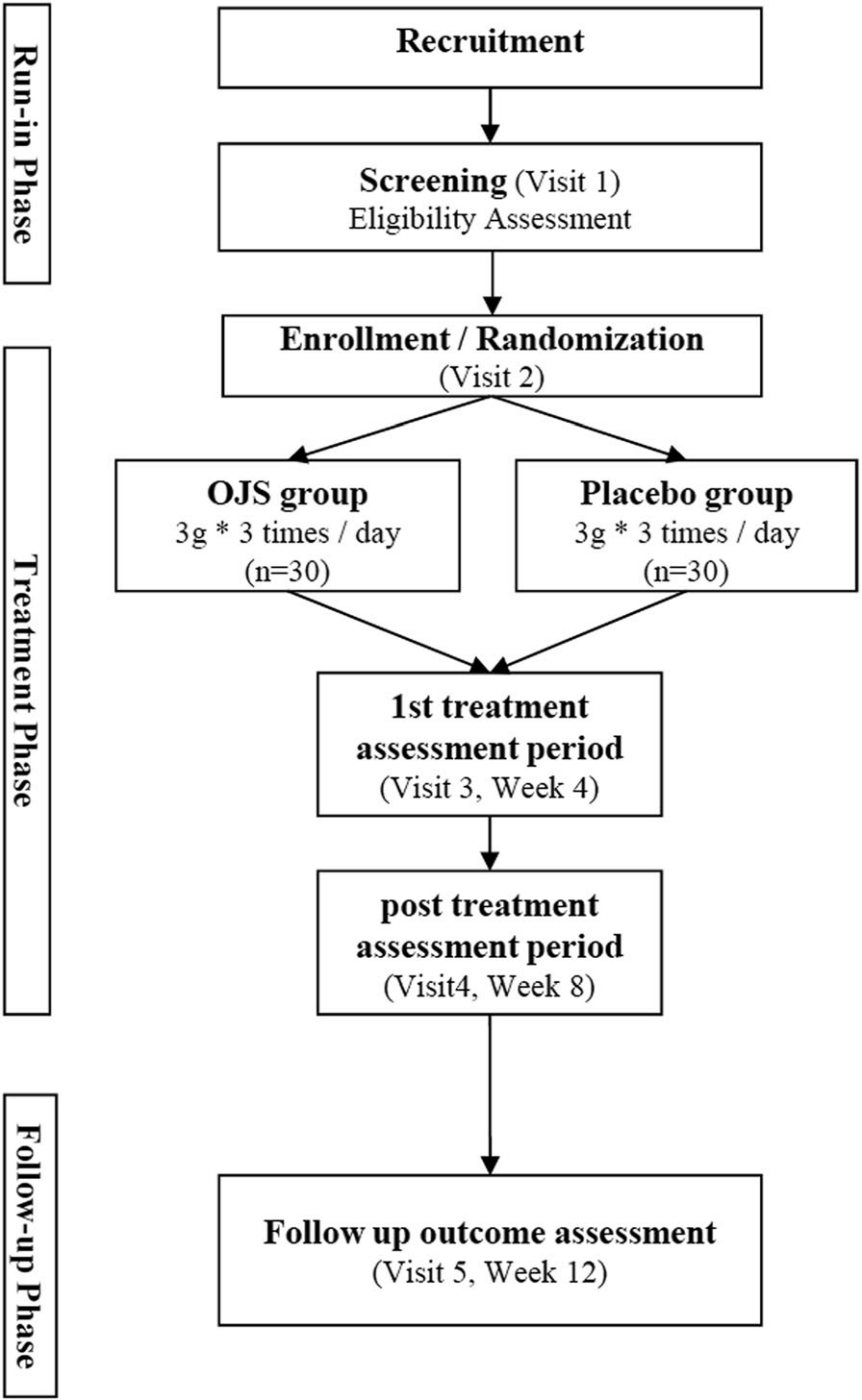
Flow chart of study procedure

This trial is a randomized, double-blinded, parallel-group, placebo-controlled, multicenter, pilot study that will be conducted at the following sites: Semyung University second affiliated Oriental Medical Hospital at Chungju, Kyung Hee University Oriental Medical Center, and Kyung Hee University Oriental Medicine Hospital at Gangdong. The total duration of study period is approximately 13 weeks from the screening visit (Fig. 1). The protocol design is based on the Consolidated Standards of Reporting Trials (CONSORT) guidelines and Standard Protocol Items: Recommendations for Interventional Trials (SPIRIT) Checklist (see Additional file 1).

A total of 60 participants, who voluntarily confirm their willingness to participate in this trial, will be recruited. Written informed consent will be obtained after giving sufficient explanation and period of time for thoughtful decision. Once the participant agrees to participate in this trial, they will be asked to undergo several medical checkups for identifying ineligible participants. After 1 week of run-in period, qualified eligible participants will be randomly assigned to the OJS or the placebo group. They will be administered the investigational products (IP) for 8 weeks and one follow-up visit will be scheduled at 4 weeks after discontinuation of medication (Table 1). The participants will be asked to return any unused investigational drugs at every visit to monitor adherence in order to calculate compliance. During the trial, the participants will be prohibited from receiving other weight-loss treatment.

**Table 1:** The detailed trial schedule

		Study period				
		Screening	Treatment period			Follow-up period
Visit		visit 1 (D − 7~ − 2)	visit 2 (D 1)	visit 3 (D 28)	visit 4 (D 56)	FU1 (D 84)
Informed consent		●				
Eligibility assessment		●				
Random allocation			●			
Socio-demographic investigation^a^		●				
Medical and drug history^b^		●				
IP distribution	OJS		●	●		
	Placebo		●	●		
Assessment						
General physical examination		●	●	●	●	●
Severity of CHHF (VAS)		●	●	●	●	●
Skin temperature measurement^c^		●	●	●	●	●
Quality of life (WHOQOL-BREF)			●	●	●	●
Cold stress test			●		●	
Vital signs		●	●	●	●	●
*Anthropometric variables*		●	●	●	●	
Chest x-ray and EKG		●				
Clinical pathological laboratory test		●			●	
Medical and drug history check-up			●	●	●	●
Adverse event check-up				●	●	●
Compliance check-up				●	●	
Blind assessment					●	

^a^Sex, date of birth, age, level of education, occupation, marital status, diet, exercise, smoking, drinking, sleeping condition, motivation of participation, etc.

^b^Including medical and drug history of CHHF

^c^Skin temperature of palm (PC9) and anterior upper arm (LU4) will be measured at the screening visit; skin temperature of thigh (ST32) and instep of foot (LR3) will also be collected additionally at visit 2

Abbreviations: *CHHF* cold hypersensitivity in the hands and feet, *EKG* electrocardiogram, *IP* investigational products, *OJS ojeok-san*, *VAS* Visual Analog Scale, *WHOQOL-BREF* WHO Quality of Life abbreviated version

### Randomization

An independent professional statistician from the contract research organization (CRO), the Institute of Safety, Efficacy, and Effectiveness Evaluation for Korean Medicine (ISEE), will be created with a block size of 2 or 4 and hospital stratification by using SAS (version 6.1.; SAS Institute Inc., Cary, NC, USA). The ISEE also develops and controls the web-based randomization system with an allocation ratio of 1:1. Randomization number assignment via the web-based system will be performed at visit 2 by site the clinical research coordinator (CRC) or research assistants. The randomization sequence table will be kept in the opaque sealed envelope by the CRO and should be opened according to Standard Operating Procedures (SOPs).

### Blinding

During the trial, clinical trial staff (investigator, CRC, research assistants) and the participant will be blinded to the type of drugs, which are randomly assigned by the web-based randomization system. In an emergency medical situation, such as, a serious adverse event (AE), unblinding will proceed according to the pre-defined plans by the CRO.

### Sample size calculation

Sample size will be not calculated because of the lack of research on the use of OJS in the treatment of patients with CHHF and pilot clinical trial for OJS. Considering visit frequency of CHHF patients and availability of workforce at each site, a total of 60 participants, 30 per group will be included.

### Participants

This study will enroll 60 patients with a complaint of CHHF, who meet the criteria shown in Table 2.

**Table 2:** Eligible criteria

Inclusion criteria	
1. Female participants aged 19 to 59 years who complain of cold hypersensitivity in the hands and feet (CHHF) 2. Patients must have at least one of the following symptoms: (a) Symptoms of CHHF at a temperature at which most individuals feel no cold (b) Symptoms of extremely cold hands and feet upon exposure to cold (c) Continuance of symptoms of cold hands upon return to a warmer environment 3. Those with a CHHF Visual Analog Scale (VAS) score of 4 or higher 4. Those for whom the temperature of the palm (PC8) is lower than that of the upper arm (LU4) by 0.3 °C or more 5. Those who can comply with all study-related procedures, medications, and evaluations 6. Those who have completed a written informed consent form	
Exclusion criteria	
1. Those taking calcium antagonists or beta-blockers for the purpose of treating CHHF 2. Those with one or more ulcers or gangrene of the finger 3. Those who have been diagnosed with hypothyroidism or are taking thyroid-related medication 4. Those who are diagnosed with an autoimmune disease 5. Those who are diagnosed with carpal tunnel syndrome or have a positive result in Tinel and Phalen’s tests 6. Those who have been diagnosed with cervical disk herniation 7. Those who have been diagnosed with diabetes 8. Those who are currently medicated with drugs that may affect CHHF symptoms, such as anticoagulants 9. Those who have serious liver dysfunction (AST and ALT, each 100 IU/L or more) or renal dysfunction (creatinine (Cr) 2.0 mg/dL or more) 10. Those who do not (cannot) comply with treatment and follow-up due to mental illness such as behavior disorder, depression, anxiety neurosis, schizophrenia, or serious mental illness 11. Those who are diagnosed with moderate anemia and hematologic disorders (adult non-pregnant women hemoglobin (Hb) level less than 7 g/dL, hematocrit (Hct) level less than 26%, white blood cell (WBC) level 11,000/mm^3^ or more) 12. Those with systolic blood pressure (SBP) 180 mmHg or diastolic blood pressure (DBP) greater than 100 mmHg based on average value of at least 2 measurements 13. Those who have suspected arrhythmia requiring treatment on EKG, or have been diagnosed with heart diseases, such as, ischemic heart disease and so on 14. Those who are addicted to alcohol or drugs 15. Fertile women who are pregnant (positive urine-HCG), have a pregnancy plan, or do not agree to the appropriate method of contraception 16, Those who have been diagnosed with a malignant tumor 17. Those who are currently participating in other clinical trials 18. Those who are unable to understand and speak Korean 19. Those who are judged to be inappropriate for the clinical study by the researchers	

### Withdrawals

Voluntary participation and informed consent is mandatory for this trial before enrollment. All participants have the right to discontinue the study at any time. The detailed withdrawal criteria in this trial are as follows: occurrence of a serious AE either due to IP or other reasons; detection of a systemic disease that was not discovered at the screening stage; less than 70% compliance; subject’s withdrawal of consent; use of any forbidden medication or treatments (anticoagulants, psychotropic drugs, antidepressants, antithyroid medications, red ginseng, and other medications or treatments that may affect CHHF) during the trial that could affect the study result; subject who cannot be followed up. Reasons for withdrawal will be documented in case report forms (CRF) and data will be analyzed using the intention-to-treat (ITT) principle. For ensuring the retention of participants, each site research coordinator will responsible for making next visit-reminder phone calls.

### Recruitment

A total of 60 participants who meet the eligible criteria will be competitively recruited from three sites of university-affiliated Korean medicine teaching hospitals. Patient recruitment will be advertised by using posters and institution website bulletin boards in trial hospitals or local community newspaper advertisements.

### Interventions

On enrollment in the study, participants will be randomly assigned to the OJS and placebo groups. Each will be administered either 3 g of OJS or the placebo drug (granulated extract) three times a day orally for 8 weeks.

OJS contains *Atractylodis Rhizoma* 44.3 mg, *Ephedrae Herba* 22.3 mg, *Citri Reticulatae Pericarpium* 22.3 mg, *Pinelliae Rhizoma* 22.3 mg, *Zingiber officinale Rosc*. 22.3 mg, *Cinnamomi Ramulus* 22.3 mg, *Angelicae Gigantis Radix* 22.3 mg, *Jujubae Fructus* 22.3 mg, *Radix Glycyrrhizae* 22.3 mg, *Paeoniae Radix Alba* 22.3 mg, *Angelicae Dahuricae Radix* 22.3 mg, *Poria (Hoelen)* 22.3 mg, *Aaurantii Immaturus Fructus* 22.3 mg, *Cnidii Rhizoma* 22.3 mg, *Magnoliae Cortex* 22.3 mg, *Platycodi Radix* 22.3 mg, and *Cyperi Rhizoma* 13.3 mg. The placebo drug will be prepared identically in all respects to the active oral drug without active ingredients, containing lactose 1.7 g, corn starch 0.1 g, citric acid, 0.1 g, OJS herbal flavor 0.1 g, and caramel food coloring 0.1 g.

Both IP will be manufactured by Hanpoong Pharm and Foods Co., Ltd. (Jeonju, Korea). The daily dosage of OJS in this trial is determined based on the information from product approval certification by the Korean Ministry of Food and Drug Safety (MFDS).

During the study period, only study IP is allowed to use. If participants are willing to administer the drugs other than IP, they should notify the site investigator prior to administration and confirm whether it is available or not.

### Outcomes

All of the assessments will be performed by the trained investigators in each site. Before initiating the trial, all of the investigators will participate in the trial SOP training for being thoroughly familiar with the trial process and outcome assessments.

#### Primary outcome variable

##### Visual Analog Scale (VAS)

The mean changes of VAS score from baseline will be assessed the severity of CHHF symptoms by using a VAS [15, 16]. It is a 10-point scale ranging from 0 (no coldness) to 10 (most severe coldness). Patients will be asked to mark on the line at a point that represents the severity of the CHHF at every visit. The higher points represent greater severity of CHHF.

#### Secondary outcome variables

The secondary outcomes include the mean changes in body skin temperature at particular acupoints, total score of the Korean version of the WHO Quality of Life-BREF.

##### Body skin temperature

The enrolled participants will be advised to avoid consuming caffeine, alcohol, tobacco, undergoing vigorous exercise, and taking hot showers for at least 2 h before clinic visit. After arrival at the clinic, the CRC seats the participants in the examination room (24 ± 1 °C) for at least 20 min for relaxation. Body skin temperature (BT) will be measured at specific acupoints of the body which are distributed on the extremities: center of both palms (PC9), anterior upper arms (LU4), thighs (ST32), and junctions of the first and second metatarsal bones (LR3) by using a thermometer (Testo 835-T1, Lenzkirch, Germany) at each visit [17]. The differences between each acupoint and two points on each of the upper and lower extremities from baseline will be calculated to evaluate the change of body temperature.

##### Cold stress test

The cold stress test (CST) is generally used to assess the severity of cold sensitivity in patients with vascular disorders [18]. For performing the procedure, the participants will rest for 20 min in the examination room (24 ± 1 °C) and then immerse both hands into a container of iced water (20 °C) for 30 s. The BTs of both palms (PC8) are assessed by using an infrared thermometer (Testo 835-T1, Lenzkirch, Germany), pre and post immersion and 6 min post immersion. The CST will be performed at visits 2 and 5. The recovery rate (RR) equation of the CST is as follows:

$$ RR=\left({T}_6-{T}_0\right)/\left({T}_{\mathrm{base}}-{T}_0\right)\times 100\%, $$

where: *T*_base_: the skin temperature of PC8 at pre immersion

*T*_0_: the skin temperature of PC8 measured at post immersion

*T*_6_: the skin temperature of PC8 measured at 6 min post immersion

The higher RR represents faster recovery to normal temperature. The mean change of RR from baseline will be calculated to evaluate the efficacy of OJS.

##### Korean version of the WHO Quality of Life-BREF

The mean changes of the WHO Quality of Life abbreviated version (WHOQOL-BREF) score from baseline will be assessed the quality of life of patients with CHHF symptoms. The WHOQOL-BREF is an effective, cross-culturally comparable, quality of life questionnaire. It consists of 26 items with five domains: general quality of life (2), physical health (7), psychological health (6), social relationships (3), and environmental health (8). To estimate quality of life scores in the Korean population, the Korean version of WHOQOL-BREF by Min et al. [19], will be used and measured at visits 2 and 4. Raw scores in each domain will be converted into a 0–100 transformed score where a higher score indicates better quality of life.

### Safety assessment

For safety assessment, we will perform the general physical examination and vital sign measurement at every visit. The kidney and liver function tests (measurement of blood urea nitrogen (BUN), creatinine (Cr), ALT, AST, and γ-GT levels) will be conducted at the screening visit and visit 4.

### Adverse event (AE) reporting

All participants will voluntarily report occurrences of AEs during trial at every visit. If there is an AE, the site investigator will immediately report to the sponsor any serious AE, whether or not considered drug related, and also report to the site Institutional Review Boards (IRB). After receiving an AE report, the site principal investigator (PI) decides whether to withdraw from or continue to participate in the trial. The collected AEs must be recorded in the CRF by the site investigator.

### Statistical analysis

An independent statistics expert will perform the statistical analysis in a blind manner. Intention-to-treat (ITT) analysis will be the main analysis for efficacy assessment for use in all participants who receive at least one dose of any IP; in addition, per-protocol (PP) analysis will be performed in participants who complete the trial except dropouts. Missing data will be adjusted using the last-observation-carried-forward (LOCF) imputation method. The statistical level of significance will be set at *P* < 0.05. All statistical analyses will be performed using SPSS, version 25.0. (IBM Inc., Chicago, IL, USA).

### General characteristics

Descriptive statistics are used to summarize the study populations and safety data. For the purposes of this analysis, descriptive statistics refers to the sample size (*N*), mean, and standard deviation for any group variable.

### Efficacy

The primary endpoint is the mean change in VAS score between the OJS group and the placebo group from baseline to V5; the secondary endpoints are the mean changes from baseline at V5 in BT, WHOQOL-BREF, and CST. Both assessments will be analyzed using chi-square or Fisher’s exact tests for categorical data and Student’s *t* tests or Mann-Whitney *U* tests for continuous data. Additionally, the repeated measures analysis of variance (ANOVA) will be performed to assess the presence of any significant differences in the mean VAS, BT, and WHOQOL-BREF scores between groups at different time points (each visit after baseline assessment).

### Safety

Safety variables will be assessed based on the frequency and severity of treatment-emergent AEs as well as changes in vital signs and laboratory parameters. Vital signs and laboratory measurements will be summarized descriptively by treatment group using both observed values and change from baseline values.

### Data management and monitoring

All records will be collected in paper CRF files. The CRFs are stored in a securely locked location. To protect confidentiality, identification information will be deleted from all study documents. Once the trial is completed, the raw data will be using a double independent entry to promote the data quality. After finishing the data entry, the database will be locked and analyzed by an independent statistician under the confirmation of the PI. Site investigators will have direct access to the datasets from their own sites.

Routine monitoring visits will be conducted by the ISEE. They will confirm whether the records of each CRF are accurate by comparing it to the source documents and whether the procedures of the trial follow the approved protocol and SOPs. No auditing will be performed for this trial.

### Ethical consideration and dissemination

The IRB of Kyung Hee University Oriental Medical Center (KOMCIRB-170120-HR-001-08), Kyung Hee University Oriental Medicine Hospital at Gangdong (2017–02–002-001), and Semyung University second affiliated Oriental Medical Hospital at Chungju (1702–03) have all approved the study. The study will be conducted in accordance the amended Declaration of Helsinki and the regulations of the “Good Clinical Practice” principles in the Korea Food and Drug Administration (KFDA). We will provide enough explanation and time for participants to decide whether to participate in the clinical study, and written informed consent will be obtained from all participants before the beginning of the clinical trial. All the personal information collected during the trial procedure will be stored as coded identification numbers for protecting participants’ confidentiality. If any protocol modification which may have impacted to the performance of the trial is needed, it should be approved by the site IRB prior to implementation. After approval, all staffs must be notified and trained before conducting the trial.

The results of the trial will be disseminated through scientific journals or presentations at scientific conferences. So far, public access to any trial related data remains unplanned.

## Discussion

CHHF is one of the most common discomforts that women experience year-round in East Asian countries [20]. While few researchers in the medical sector have tried to investigate the mechanism of the condition, diagnosis and treatment, it is still not standardized systematically.

The herbal prescription, OJS, has been widely prescribed clinically in various cold-induced symptoms in TKM [21]. Since scientific evidence for treatment of patients with CHHF is lacking, we organized an experts’ meeting consisting of 10 TKM experts, each with clinical experience of more than 10 years, and with experience of treating patients with CHHF to determine treatment efficacy of OJS in patients with CHHF. Based on the expert consensus, *Danggui-Sayuk-Ga-Osuyu-Saenggang-tang* (DSGOST), OJS, and *Sipjeondaebo-Tang* (SJDBT) are the most frequently prescribed medications for treating patients with CHHF in TKM hospitals. Thus, our research team is also working on a series of pilot clinical trials of herbal medicine to define the general characteristics of conditions and the efficacy of herbal medicine in patients with CHHF. Among the most prescribed medication, a trial of DSGOST has been performed in several TKM hospitals and was completed in 2017; the results will be published in peer-review journals [22]. In the second trial in the series, the OJS trial protocol is revised based on the troubleshooting ideas from the DSGOST trial to provide convenient and sufficient procedures to trial staff. The initiation of the OJS trial was in 2017 and enrollment remains ongoing.

The strength of this study is that it is the first well-designed RCT to evaluate the efficacy and safety of OJS compared to placebo in patients with CHHF in Korea. The findings from the studies should provide meaningful information to design a confirmative, large-scale RCT in the near future as well as supporting evidence of clinical practice.

This trial has few limitations. First, there is an absence of age stratification in participant enrollment. As this is a small-scale exploratory study, various unique characteristics due to female lifecycle changes, such as reproductive factors [23], are unable to be considered in this trial. In addition, the total number of patients enrolled is restricted by possible enrollments in each of the participating institutions. Thus, we will consider adapting the stratification to investigate CHHF in different age groups in a further large-scale study in the near future. The other limitation is the lack of performing laboratory tests to rule out various CHH-related diseases. So far, the pathophysiology of CHHF has not been completely clarified; hence, we could not specify the exclusion criteria. However, we will perform physical examinations and several laboratory tests to exclude the patients with suspected secondary diseases.

## Trial status

Participant recruitment began on 20 March 2017 and 50 participants have been recruited until now.

## Additional file

Additional file 1: Standard Protocol Items: Recommendations for Interventional Trials **(**SPIRIT) 2013 Checklist: recommended items to address in a clinical trial protocol and related documents^*^. (PDF 39 kb)

## Abbreviations

AE: Adverse event
ALT: Alanine aminotransferase
ANOVA: Analysis of variance
AST: Aspartate aminotransferase
BT: Body skin temperature
BUN: Blood urea nitrogen
CHHF: Cold hypersensitivity in the hands and feet
CM: Conventional medicine
CRC: Clinical research coordinator
CRF: Case report form
CRO: Contract research organization
CST: Cold stress test
IP: Investigational products
IRB: Institutional Review Boards
ISEE: Institute of Safety, Efficacy, and Effectiveness Evaluation for Korean Medicine
ITT: Intention to treat
KFDA: The Korea Food and Drug Administration
LOCF: Last-observation-carried-forward
NHI: National Health Insurance
OJS: *Ojeok-san*
PP: Per protocol
RCT: Randomized clinical trial
RP: Raynaud’s phenomenon
RR: Recovery rate
SOPs: Standard Operating Procedures
TCM: Traditional Chinese medicine
TKM: Traditional Korean medicine
VAS: Visual Analog Scale
WHOQOL-BREF: WHO Quality of Life Scale abbreviated version
γ-GT: Gamma glutamyl transferase
